# Viral-Targeted Strategies Against EBV-Associated Lymphoproliferative Diseases

**DOI:** 10.3389/fonc.2019.00081

**Published:** 2019-02-26

**Authors:** Kwai Fung Hui, Stephanie Pei Tung Yiu, Kam Pui Tam, Alan Kwok Shing Chiang

**Affiliations:** ^1^Department of Paediatrics and Adolescent Medicine, Li Ka Shing Faculty of Medicine, Queen Mary Hospital, The University of Hong Kong, Hong Kong, Hong Kong; ^2^Center for Nasopharyngeal Carcinoma Research, The University of Hong Kong, Hong Kong, Hong Kong

**Keywords:** Epstein-Barr virus, lymphoproliferative diseases, viral-targeted strategies, EBV latency, lytic cycle reactivation, histone deacetylase inhibitors, proteasome inhibitors

## Abstract

Epstein-Barr virus (EBV) is strongly associated with a spectrum of EBV-associated lymphoproliferative diseases (EBV-LPDs) ranging from post-transplant lymphoproliferative disorder, B cell lymphomas (e.g., endemic Burkitt lymphoma, Hodgkin lymphoma, and diffuse large B cell lymphoma) to NK or T cell lymphoma (e.g., nasal NK/T-cell lymphoma). The virus expresses a number of latent viral proteins which are able to manipulate cell cycle and cell death processes to promote survival of the tumor cells. Several FDA-approved drugs or novel compounds have been shown to induce killing of some of the EBV-LPDs by inhibiting the function of latent viral proteins or activating the viral lytic cycle from latency. Here, we aim to provide an overview on the mechanisms by which EBV employs to drive the pathogenesis of various EBV-LPDs and to maintain the survival of the tumor cells followed by a discussion on the development of viral-targeted strategies based on the understanding of the patho-mechanisms.

## Introduction

Epstein-Barr virus (EBV) is a ubiquitous gamma herpesvirus which establishes life-long persistence in 90% of the human populations (1). This virus is closely associated with nasopharyngeal carcinoma (NPC), a subset of gastric carcinoma and several types of lymphoproliferative diseases (LPDs), such as endemic Burkitt lymphoma (BL), Hodgkin lymphoma (HL), nasal NK/T-cell lymphoma, diffuse large B cell lymphoma (DLBCL), AIDS-associated B-cell lymphoma and post-transplant lymphoproliferative disorder (PTLD) (2, 3). EBV is shown to transform primary B cells and could contribute to the pathogenesis of EBV-LPDs *in vitro*. In these cancer cells, EBV usually persists in a tightly latent state to escape from the human immune surveillance. Occasionally, the virus can switch from the latent cycle to the lytic cycle in response to various physiologic stimuli. At various pathogenic stages of EBV-LPDs, the virus expresses a number of viral latent or lytic proteins to manipulate cell cycle and cell death processes and promote the survival of the tumor cells. This review will summarize the pathogenic mechanisms which are affected by the EBV latent and lytic proteins for the survival of-EBV-LPDs and discuss on the development of therapeutic strategies targeting the patho-mechanisms associated with EBV-LPDs.

## EBV Latency in EBV-LPDs

**Figure 1:**
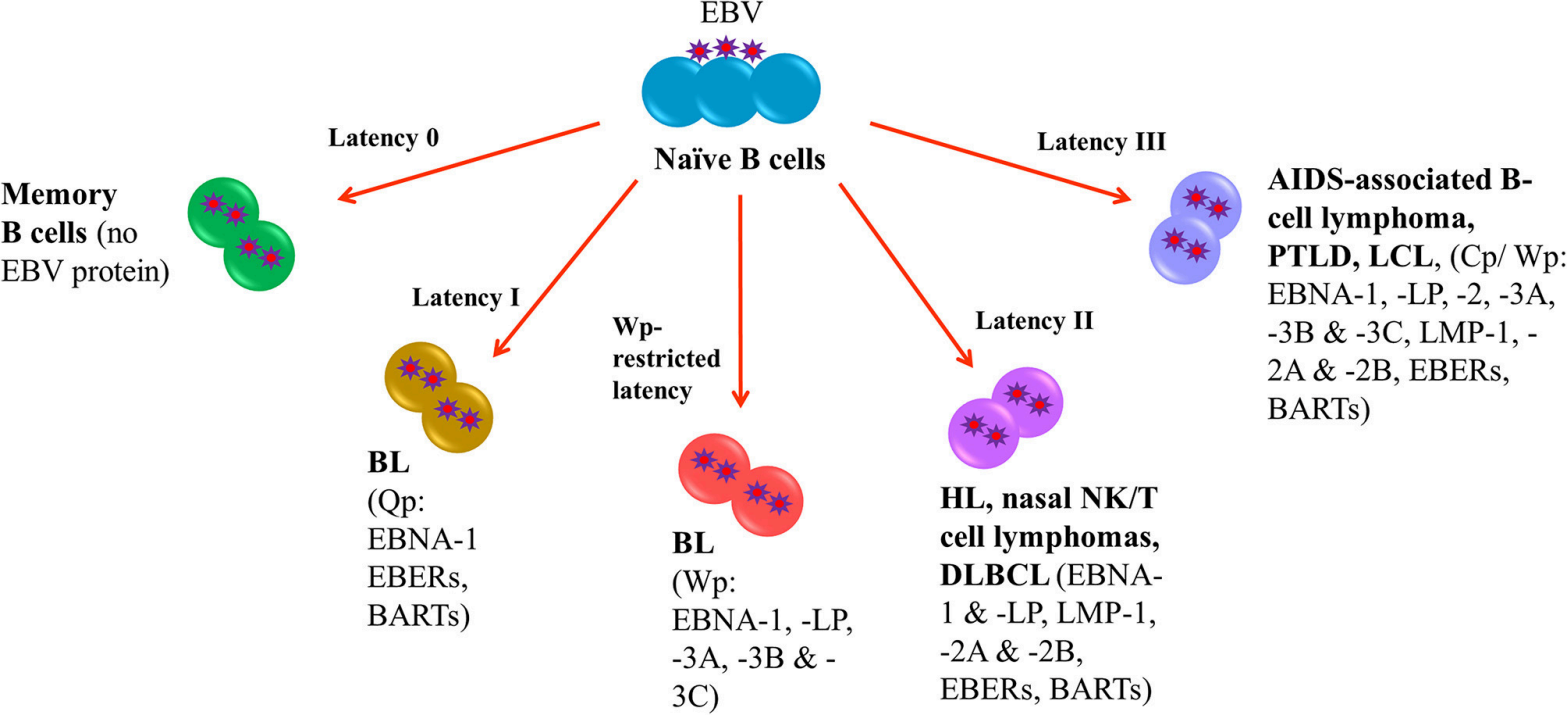
EBV latency in EBV-LPDs. No EBV protein is expressed in Latency 0. Only EBNA-1, EBERs, and BARTs are expressed in Latency I which is associated with endemic BL. The transcription of EBNA-1 is initiated at the BamHI Q promoter. 15% of endemic BL is found to be Wp-restricted latency in which EBNA-LP, EBNA-1, EBNA-3A, -3B, and -3C are transcribed from the BamHI W promoter. HL, nasal NK/T-cell lymphoma and DLBCL are detected in type II latency that EBNA-1, EBNA-LP, latent membrane protein (LMP)-1, -2A, and -2B, EBERs and BARTs are expressed. AIDS-associated B-cell lymphoma, PTLD and lymphoblastoid cell line (LCL), an *in vitro* model of EBV-LPDs are observed in type III latency. All EBV nuclear antigens (EBNA-1, -2, -LP, -3A, -3B, and -3C), latent membrane proteins (LMP-1, -2A, and -2B), EBERs and BARTs are expressed.

Following EBV infection, the virus is able to establish life-long infection in memory B cells where no EBV protein is expressed (latency 0). In EBV-LPDs, the virus can express four different latency patterns, namely, type I, type II, type III and Wp-restricted latency as characterized by the expression patterns of EBV latent proteins. In type I latency, which is observed in the majority of endemic BL, the expression of viral genes is greatly restricted with only EBV nuclear antigen (EBNA)-1, EBV-encoded small RNAs (EBERs) and BamHI-A rightward transcripts (BARTs) are expressed. The transcription of EBNA-1 is initiated at the BamHI Q promoter (Qp) (4). In addition to type I latency, Wp-restricted latency could also be detected in ~15% of the endemic BL (5). In this latency, EBNA-LP, EBNA-1, EBNA-3A, -3B, and -3C are transcribed from the BamHI W promoter (Wp) (6). In type II latency, which is observed in HL, nasal NK/T-cell lymphoma and DLBCL, more latent genes including EBNA-1, EBNA-LP, latent membrane protein (LMP)-1, -2A, and -2B, EBERs and BARTs are expressed. Type III latency is detected in AIDS-associated B-cell lymphoma, PTLD and lymphoblastoid cell line (LCL), an *in vitro* model of EBV-LPDs. This is the most immunogenic form of latency in which a full set of latent genes including EBNA-1, -2, -LP, -3A, -3B, -3C, LMP-1, -2A, -2B, EBERs and BARTs are expressed (6, 7). Either BamHI C promoter (Cp) or Wp is activated to drive the expression of the EBV latent genes in this latency (Figure 1).

## EBV Lytic Replication

**Figure 2:**
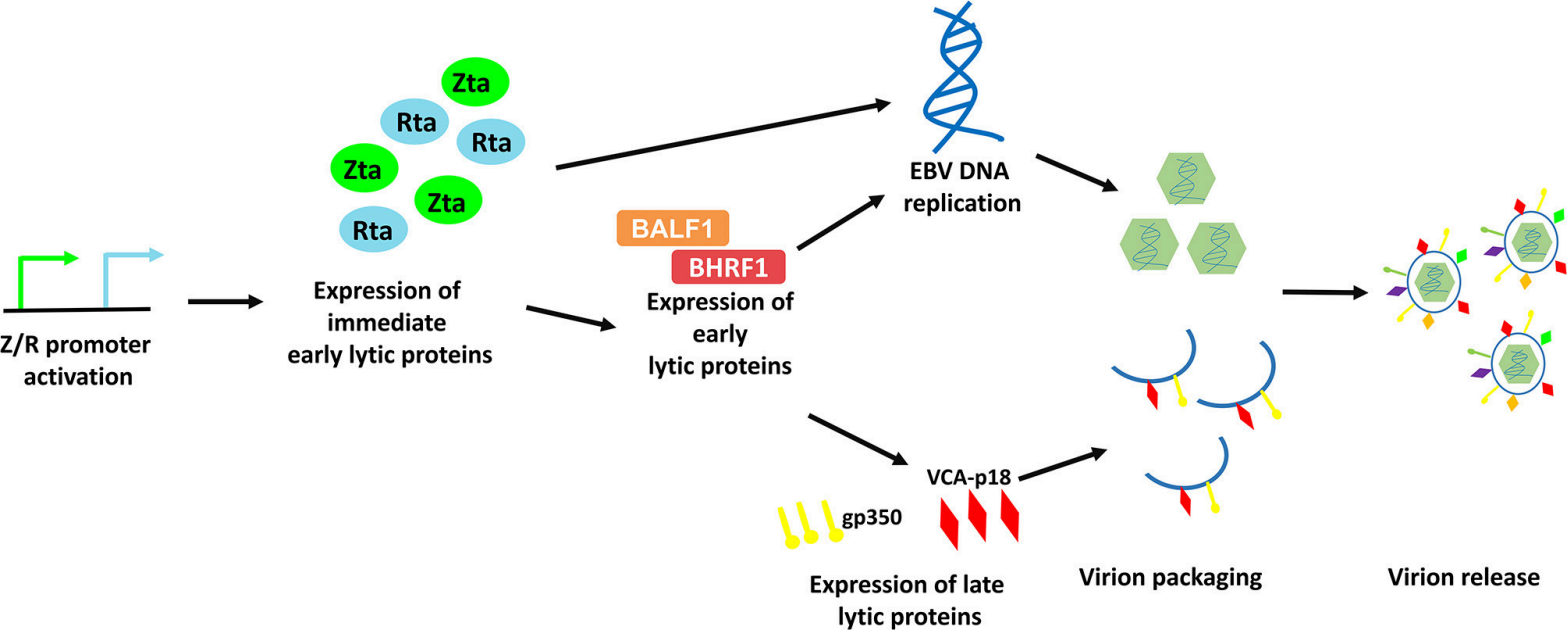
Schematic diagram representing the sequential events occur during EBV lytic reactivation. EBV Z/R promoters are activated upon diverse stimulants e.g., B-cell receptor crosslinking, chemical inductions and cellular stresses, resulting in the expression of immediate early lytic proteins, Zta and Rta. These key drivers of EBV lytic reactivation subsequently induce EBV viral DNA replication and the expression of an array of viral lytic proteins including early lytic proteins e.g., BALF1 and BHRF1 and late lytic proteins e.g., gp350 and VCA-p18. Viral DNA is then being packaged with the help from structural proteins and is assembled into mature virion. Finally, EBV is released via exocytosis.

EBV lytic cycle reactivation has been comprehensively studied in the Akata BL cell line, in which the lytic cycle of EBV can be efficiently induced by cross-linking the cell surface receptor with anti-human IgG antibody (8). This model provides an effective way to study the possible physiological mechanisms of viral lytic reactivation in EBV-LPDs. EBV lytic cycle is initiated with the expression of two immediate early proteins, namely Zta and Rta (9–11). Expression of these two immediate early proteins activates the expression of one another and subsequently triggers the expression of a panel of early lytic proteins (e.g., BMRF1, BALF1, BHRF1, etc.,) (3, 12). EBV immediate early and early lytic proteins initiate viral DNA replication and later, the expression of late lytic proteins (e.g., VCA-p18, gp350/220, etc.,) (3). Anti-viral drugs e.g., phosphonoformic acid, which suppress EBV DNA replication can also inhibit expression of EBV late lytic proteins, suggesting that EBV DNA replication is an upstream process that regulates late lytic protein expression (3, 13–15). In case of a complete lytic cycle, the viral DNA is replicated as large head-to-tail molecules which are then cleaved into pieces and packaged into viral progenies for dissemination to the neighboring cells (16). More than 70 EBV lytic genes, which are important for viral replication, dissemination and infection, are expressed during the EBV lytic cycle (Figure 2).

## Immunity Against EBV-LPDs

**Figure 3:**
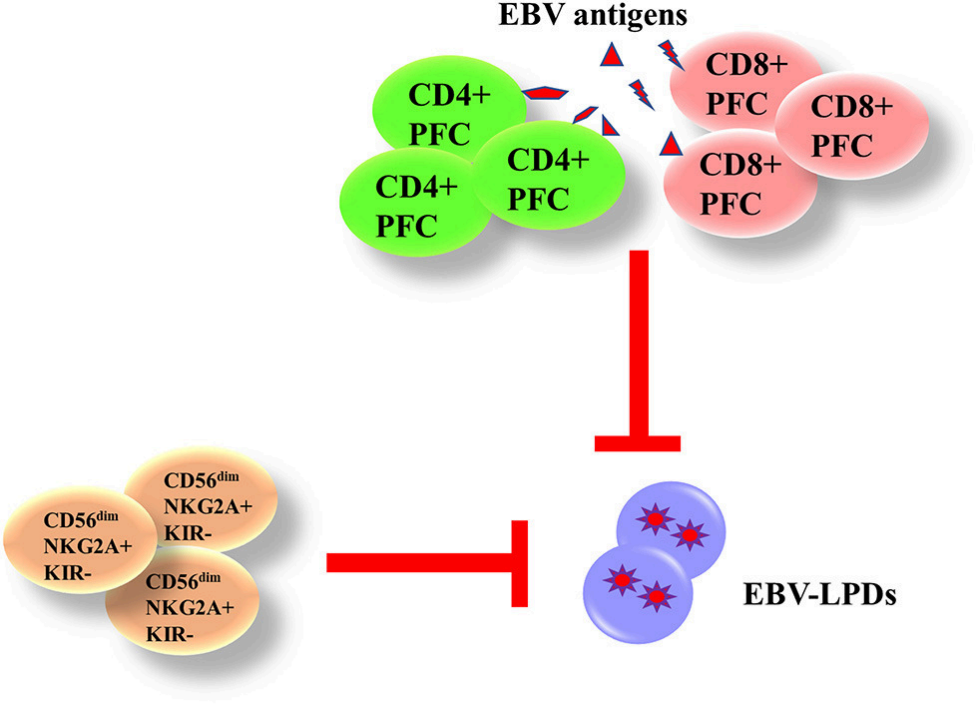
Immunity against EBV-LPDs. (IFN)-γ and other functional cytokines [(TNF)-α and IL-2] are produced from EBV-specific polyfunctional T cells (PFCs) to control the proliferation of EBV-infected B cell during long-term infection. There are increase responses of CD4^+^ and CD8^+^ PFCs in infectious mononucleosis (IM) patients. (CD56^dim^NKG2A^+^KIR^−^) NK cells also control EBV infection in acute IM patients and kill LCLs.

Both innate and adaptive immunity are responsible for the control of EBV. The phagocytes and natural killer (NK) cells in the innate immunity are responsible for the control of immediate B cell infection and virus replication. The CD4^+^ and CD8^+^ T cells in the adaptive immunity are capable of producing interferon (IFN)-γ and other functional cytokines to control the proliferation of EBV-infected B cells during long-term infection. We and others have demonstrated that the presence of EBV-specific polyfunctional T cells (PFCs), which could produce multiple cytokines [e.g., IFN-γ, tumor necrosis factor (TNF)-α, interleukin (IL)-2] simultaneously and readily degranulating, in long-term EBV carriers (17, 18). A clear increase in CD4^+^ and CD8^+^ PFC responses against EBV antigens is also demonstrated in infectious mononucleosis (IM) patients, correlating with increased cytotoxicity of T cells against autologous LCLs (19). NK cells play a complementary role with T cells in controlling tumor growths and viral infections. Azzi et al. have demonstrated that a subset of early-differentiated (CD56^dim^NKG2A^+^KIR^−^) NK cells play a more important role than the terminally differentiated (CD56^dim^NKG2A^−^KIR^+^) NK cells in the control of EBV infection in acute IM patients (20). In addition, Hatton et al. have also shown that a NKG2A-expressing subset of NK cells could effectively kill EBV-transformed autologous LCLs (21). We postulate that impairment of EBV-specific PFCs and NKG2A^+^ NK cells may contribute to the development of EBV-LPDs (Figure 3).

## Role of Latent and Lytic Viral Proteins in the Pathogenesis of EBV-LPDs

EBV effectively infects normal B cells and transforms them into proliferating LCLs *in vitro*. EBV latent proteins are shown to contribute to the pathogenesis of EBV-LPDs. Besides, there is increasing evidence that shows that EBV lytic proteins can also promote the pathogenesis of EBV-associated diseases. The viral latent and lytic proteins can maintain the proliferation and survival of EBV-positive cancer cells via deregulating the cellular mechanisms that regulate cell cycle, apoptosis and immune recognition of the host cells.

### Deregulation of Cell Cycle

**Figure 4:**
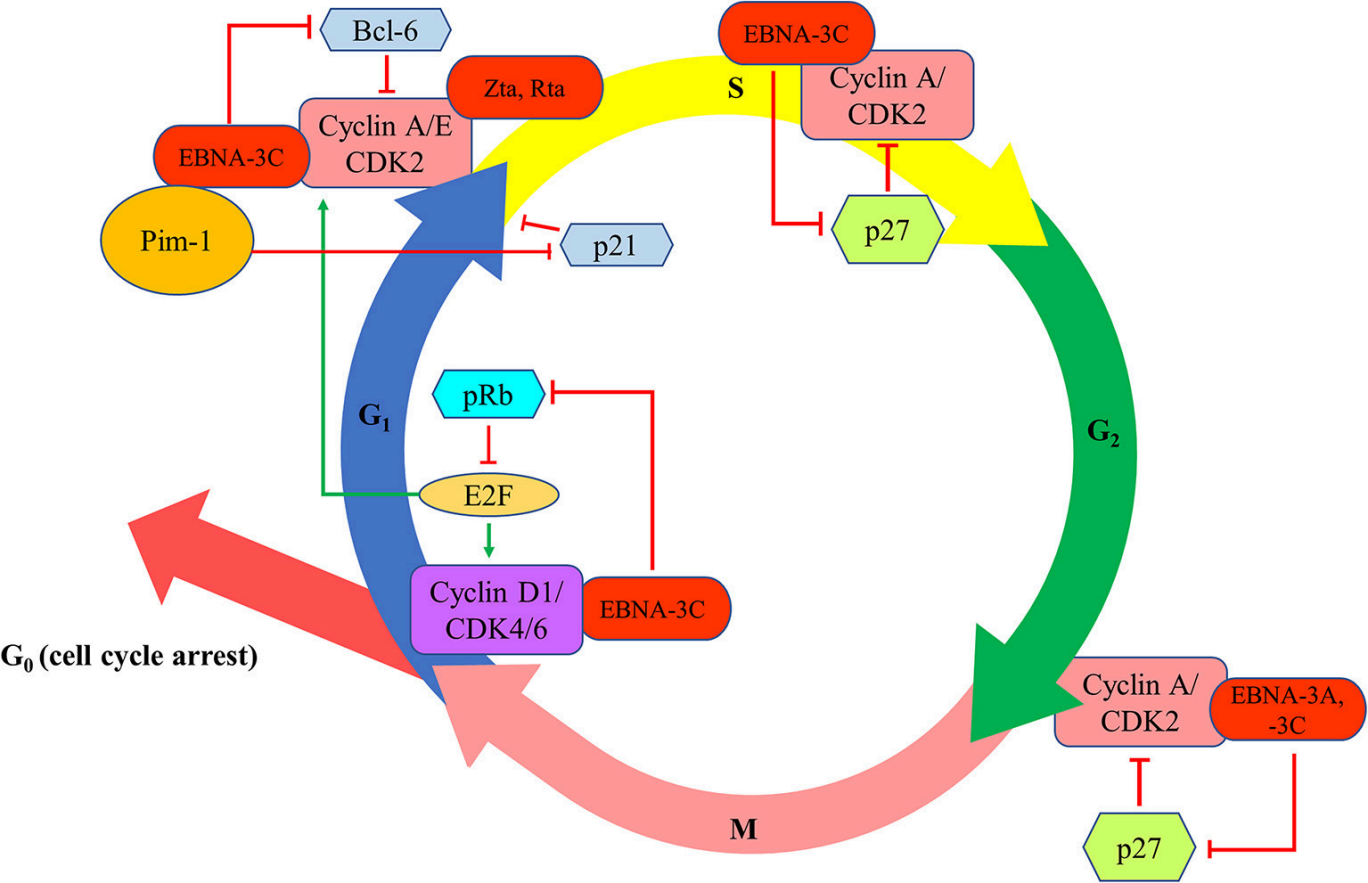
Effects of EBV latent and lytic proteins on the regulation of cell cycle. EBNA-3C interacts with cyclin A/CDK2 and promotes the proteasomal degradation of p27^KIP1^ to assist the EBV-infected cells to progress to enter S phase and M phase. EBNA-3C mediates the ubiquitin-proteasome degradation of pRb, increasing the transcription of E2F-dependent cyclin/CDK complexes (cyclin-D1/CDK-4/-6 and cyclin-A/-E/CDK-2), to allow the cells to enter G1 phase from G0 phase and enter S phase from G1 phase, respectively. EBNA-3C stabilizes Pim-1 protein to promote the phosphorylation and subsequent proteasomal degradation of p21^WAF1^ for the cells to enter S phase from G1 phase. EBNA-3C also promotes the proteasomal degradation of Bcl-6 which subsequently releases cyclin-D1 for the transition of G1 to S phase. EBNA-3A and -3C co-operate in epigenetic repression of p14^ARF^ and p16^INK4a^ to facilitate the transformation and proliferation of EBV-LPDs through bypassing the G2/M checkpoint regulation upon stimulation by various cytotoxic stresses.

EBNA-3A and -3C proteins are shown to manipulate the cell cycle of the host cells to facilitate successful transformation of B cells and to maintain the proliferation of the transformed cells. The first evidence of the cell cycle regulatory property of EBNA-3 proteins is demonstrated by Allday et al. who found that mutation of EBNA-3C can lead to G1 cell cycle arrest in B cells (22). It is further shown that EBNA-3C can directly interact with cyclin A to stimulate the activity of cyclin-dependent kinase (CDK)-2 and subsequently facilitates LCLs to pass through the retinoblastoma protein (pRb) cell cycle checkpoint (23). EBNA-3C can also stabilize cyclin A and promote the proteasomal degradation of p27^KIP1^, hence assists the EBV-infected cells to progress to M phase (24, 25). EBNA-3C also mediates the ubiquitin-proteasome degradation of pRb by recruiting SCF^Skp2^ E3-ubiquitin ligase (26, 27). Consequently, fewer pRb can interact with the transcription factors of E2F family which then suppress the transcription of E2F-dependent cyclin/CDK complexes. More E2F-dependent complexes, including the cyclin-D1/CDK-4/-6 and cyclin-A/-E/CDK-2, will be expressed to allow the cells to enter G1 phase from G0 phase and enter S phase from G1 phase, respectively (27). Furthermore, EBNA-3C also stabilizes Pim-1 protein to promote the phosphorylation and subsequent proteasomal degradation of p21^WAF1^ for the cells to enter S phase from G1 phase (28). Additionally, EBNA-3C can modulate Skp2 to mediate proteasomal degradation of p27^KIP1^ which subsequently free the cyclin-A/CDK-2 complex for the cells to enter S phase (29). EBNA-3C also promotes the proteasomal degradation of Bcl-6 which subsequently releases cyclin-D1 for the transition of G1 to S phase (30). Besides, EBNA-3A and -3C are found to co-operate in epigenetic repression of p14^ARF^ and p16^INK4a^, facilitating the transformation and proliferation of EBV-LPDs (31–33). EBNA-3A and -3C can also facilitate the EBV-LPDs to bypass the G2/M checkpoint regulation upon stimulation by various cytotoxic stresses (34–38) (Figure 4).

Reactivation of EBV lytic cycle is also shown to disrupt various cell cycle checkpoints in EBV-infected cells. Zta and Rta can promote the transition from G1 to S phase in BL cells via a mechanism related to the modulation of p53 and p21^WAF1^ (39). On the other hand, overexpression of Zta protein can induce G1 cell cycle arrest in EBV-positive cells via the interaction with CCAAT/enhancer binding proteins (C/EBP), which subsequently lead to the activation of p53 andthe accumulation of p21^WAF1^ and p27^KIP1^ (40). EBV lytic proteins can also activate the cyclin-E/CDK-2 complex for the entry of S phase in hopeto provide an environment suitable for viral DNA replication (41) (Figure 4). Interestingly, treatment of CDK inhibitors, such as purvalanol-A and roscovitine, which inhibit the transition from G1 to S phase of the cell cycle, can block lytic replication of EBV (42). Reactivation of EBV lytic cycle can also lead to G2/M arrest of the host cells. For instance, expression of Zta is shown to induce both G2/M arrest and mitotic block in HeLa cells (43); whilst treatment with 5-azacytidine (5-AZA) can lead to a G2/M phase arrest in Zta-expressing Rael cells (44). We have also reported that a histone deacetylase (HDAC) inhibitor, suberoylanilide hydroxamic acid (SAHA), can reactivate EBV lytic cycle and mediates a pronounced G2/M arrest in EBV-positive epithelial cells (11). We further showed that the induction of G2/M arrest is possibly due to the upregulation of p21^WAF1^ and downregulation of cycli-D1, p-Rb, cyclin-B1 and p-CDK-1 (45).

### Inhibition of Apoptosis

**Figure 5:**
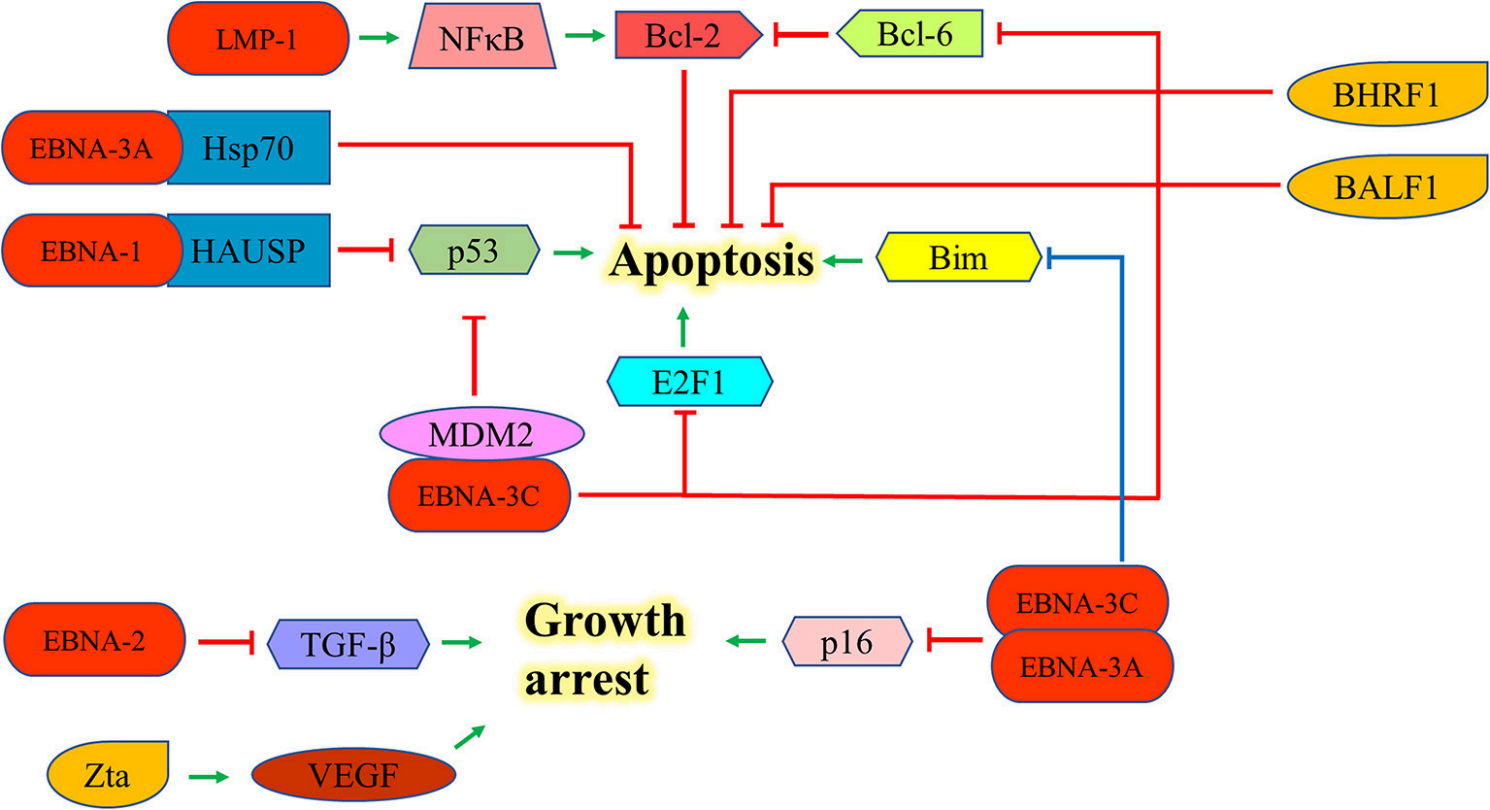
Effects of EBV latent and lytic proteins on inhibition of apoptosis. EBNA-1 interacts with the HAUSP to destabilize and degrade p53. EBNA-2 antagonizes TGF-β-mediated growth arrest in LCLs. LMP-1 upregulates Bcl-2 and promotes the growth of BL through the activation of NF-kB signaling pathway. EBNA-3A upregulates Hsp70 chaperones to suppress the apoptosis in exposure to cytotoxic agents. EBNA-3C can suppress p53-dependent apoptosis through repressing the transcription of p53 and promoting its degradation. EBNA-3C also hinders the E2F1-mediated apoptosis induced by DNA damage response through inhibiting the DNA binding activity of E2F1 and promoting its proteolysis. EBNA-3C also interacts with Bcl-6 and releases the Bcl-2 to suppress apoptosis. EBNA-3A and -3C can co-operate to repress the expression of p16^INK4a^ and Bim to promote cell proliferation. Zta can induce the expression of vascular endothelial growth factor (VEGF) to promote the growth of LCL.

EBNA-1 can interact with the herpesvirus associated ubiquitin-specific protease to destabilize and degrade p53 to inhibit apoptosis (46). EBNA-2 antagonizes TGF-β-mediated growth arrest in LCLs (47). LMP-1 also upregulates Bcl-2 and promotes the growth of BL through the activation of NF-kB signaling pathway (48). EBNA-3A upregulates Hsp70 chaperones to suppress apoptosis in exposure to cytotoxic agents (49). EBNA-3C can suppress the p53-mediated cell death by inhibiting the transcription of p53and promoting its degradation (50–52). EBNA-3C also hinders the E2F1-mediated apoptosis induced by DNA damage response throughinhibiting the DNA binding activity of E2F1 and promoting its proteolysis (53). Furthermore, EBNA-3C prevents the proteosomal degradation of MDM2 and to recruit it to initiate the degradation of p53 in order to promote the survival of EBV-LPDs (54–56). EBNA-3C also interacts with Bcl-6 and releases Bcl-2 to suppress apoptosis for lymphomagenesis (30). EBNA-3A and -3C can co-operate to repress the expression of the tumor suppressor gene, p16^INK4a^, to promote cell proliferation and prevent cell death (31–33). Moreover, they also epigenetically repress the Bim promoter which eventually suppresses the Bim-mediated intrinsic pathway of apoptosis (57, 58) (Figure 5).

Expression of EBV lytic proteins also plays a role in pathogenesis of EBV-positive cancers by inhibiting apoptotic cell death. Zta can induce the expression of vascular endothelial growth factor (VEGF) to promote the growth of LCL (59). The early lytic genes, BHRF1 and BALF1, which encode Bcl-2 homologs, can inhibit apoptosis of EBV-associated lymphoid cancers and promote the survival of cancer cells during EBV lytic replication (60, 61). Exogenous expression of BHRF1 protein was shown to protect BJAB cells from apoptotic cell death (60); whilst expression of BALF1 protein inhibits Fas ligand-induced apoptosis in HeLa cells (61) (Figure 5). Several EBV lytic genes including BALF3, BARF1, BGLF4, and BGLF5 which induces DNA damage response and genomic instability could also contribute to the carcinogenesis of EBV-associated cancers (62–65).

### Immune Evasion

**Figure 6:**
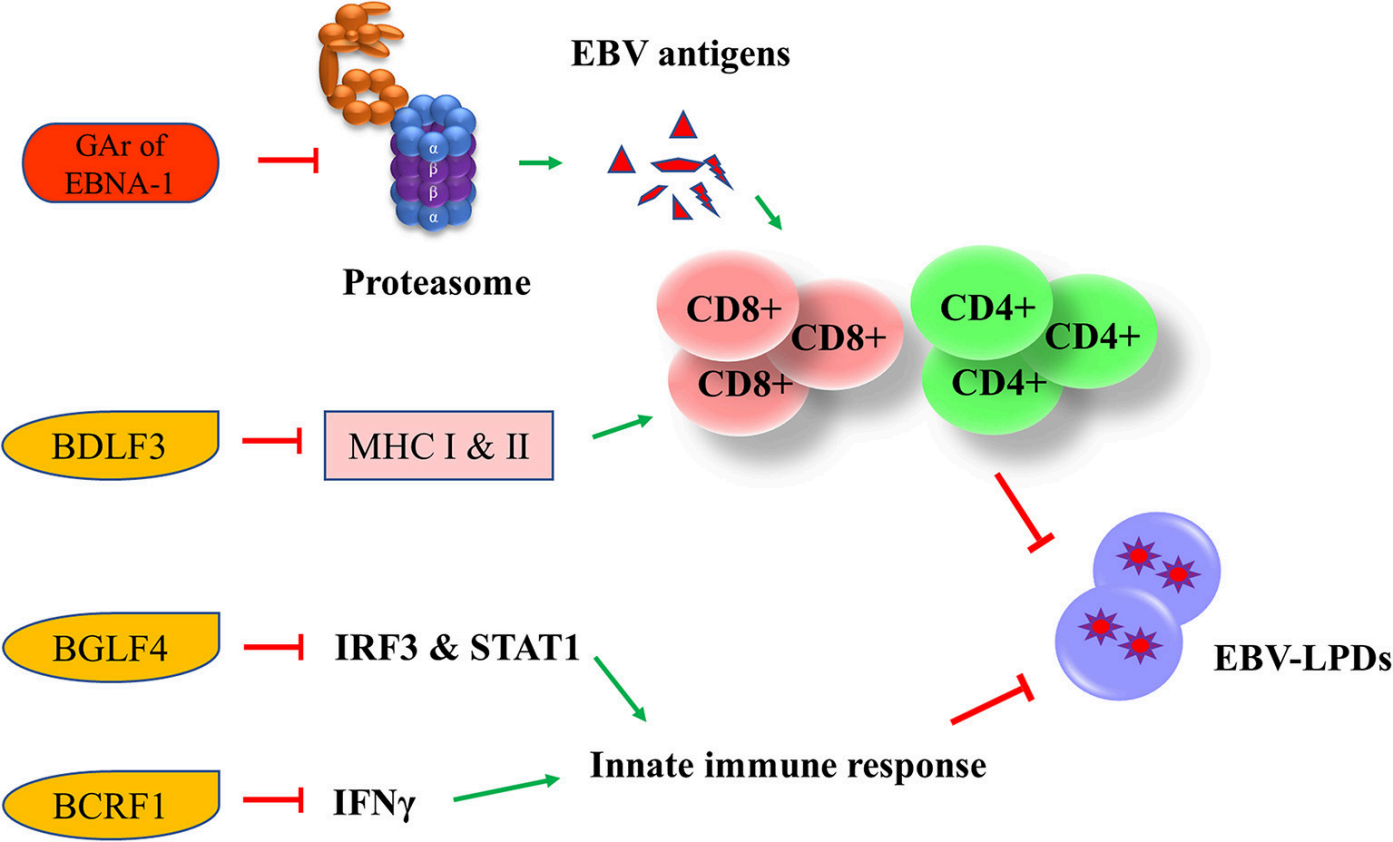
Effects of EBV latent and lytic proteins on immune evasion. Glycine-alanine repeats (GAr) of EBNA-1 render it not be processed and presented to CD8^+^ T cells via the class I MHC. BDLF3, can promote the degradation of MHC class I and II molecules, impairing the immune recognition by EBV-specific CD4^+^ and CD8^+^ T cells. BGLF4 can suppress the host innate immune through the inhibition of interferon regulatory factor 3 (IRF3) and STAT1. BCRF1 suppresses INF-γ synthesis from human peripheral blood mononuclear cells, thus allowing the tumor cells to evade from the host immune surveillance.

EBV has developed multiple strategies to escape from the human immune surveillance. In EBV-infected B cells, the presence of glycine-alanine repeats of EBNA-1 rendered it not being able to be processed and presented to CD8^+^ T cells via class I MHC (66). Zta can induce the secretion of IL-6, -8, -10, and -13 which function to promote the tumorigenesis of different EBV-associated cancers (67). An early lytic protein, BGLF4 protein kinase, can suppress the host innate immune responses and facilitates the production of viral progenies in NPC through inhibiting the interferon regulatory factor 3 and STAT1 (68). Another EBV lytic gene, BCRF1, which encodes for a homolog of cellular IL-10, suppresses INF-γ synthesis from human peripheral blood mononuclear cells, thus allowing the tumor cells to evade from the host immune surveillance (69). BCRF1 can also function as a paracrine growth factor to enhance the transformation of B cells and promote the growth of EBV-LPDs (70). A late EBV lytic gene, BDLF3, can promote the degradation of MHC class I and II molecules, thereby impairing the immune recognition by EBV-specific CD4^+^ and CD8^+^ T cells (71) (Figure 6).

## Viral-Targeted Therapies Against EBV-LPDs

EBV persists in a tightly latent state in every tumor cell in EBV-LPDs and therefore, could be served as an excellent target for therapeutic treatments. Various viral-targeted therapies targeting the expression of EBV latent and lytic proteins for the treatment of EBV-LPDs are discussed below.

### Gene Therapy

EBV-based gene therapies have been developed to deliver cytotoxic proteins or chemosensitizers to EBV-infected malignancies. Franken et al. have shown that a targeted expression of thymidine kinase in BL using an EBNA2-responsive Cp could selectively enhance the sensitivity of EBNA2-expressing cells to an anti-viral drug, ganciclovir, *in vitro*, and *in vivo* (72). An adenovirus vector with the transgene expression regulated by the origin of replication of EBV can precisely deliver p53 into EBV-positive cancer cells and induced apoptosis to the cells (73). The use of such replication-deficient adenovirus vector for EBV-targeted gene therapy is further demonstrated to be feasible *in vivo* (74).

### Immunotherapy

Several laboratories have demonstrated that the adoptive immunotherapy which employs an *ex vivo* expanded virus-specific cytotoxic T lymphocytes (CTLs) is a safe and effective treatment strategy for EBV-associated malignancies including HL, NK/T-cell lymphoma, PTLD and NPC (75, 76). Briefly, CTLs which target EBV latent proteins are isolated from patients followed by activation and expansion *in vitro* and then infused back into the patients (75). Recently, one of such autologous T cell therapies, CMD-003, has been granted fast track designation by the FDA for treating relapsed/refractory lymphoma and PTLD. Since the upregulation of PD-L1 is observed in various EBV-LPDs, another potential immunotherapy for EBV-LPDs could be by blocking the PD1 and PD-L1 pathways (77–79). Development of novel therapeutic strategy using the combination of autologous T cell therapy and PD-L1 inhibitor might potentially yield synergistic effect on the treatment of EBV-LPDs. Other immunological approaches, such as the development of vaccines and specific monoclonal antibodies against EBV are also under investigations (80). For instance, immunization with polyvalent EBV virus-like particle (VLP) vaccines (gH/gL-EBNA-1 and gB-LMP2) without adjuvant is shown to induce high neutralizing antibody titres against EBV *in vitro* and *in vivo* (81).

### Targeting EBV Lytic Cycle

Reactivation of EBV lytic cycle is another potential strategy that exploits the presence of EBV genome in tumor cells. Several reports show that reactivation of viral lytic cycle can directly induce apoptotic cell death in various EBV-infected cell lines (82–85). Kawanishi et al. have demonstrated that reactivation of EBV lytic cycle by tetradecanoyl phorbol acetate (TPA) can result in fragmentation of chromosomal DNA in Raji BL cells (82). Rta can also induce irreversible G1 arrest, cellular senescence and apoptosis in different EBV-positive cancer cell lines (86). Some of the carcinogenic lytic proteins, such as Zta and BGLF5, were reported to have contradictory roles in mediating cancer cell death. For instance, expression of Zta can phosphorylate p53 to mediate a direct killing of EBV-positive cells (43). The EBV early lytic gene, BGLF5, which encodes for EBV alkaline exonuclease, possesses a shutoff activity during lytic cycle reactivation could potentially induce apoptosis (87). We have shown that lytic cycle reactivation by HDAC inhibitors, including trichostatin A, sodium butyrate, valproic acid and SAHA, can lead to enhanced apoptosis in NPC and gastric carcinoma cells (11, 88). The induction of apoptosis is mediated through the upregulation of p21^WAF1^ and cell cycle arrest at G2/M phase (45).

**Figure 7:**
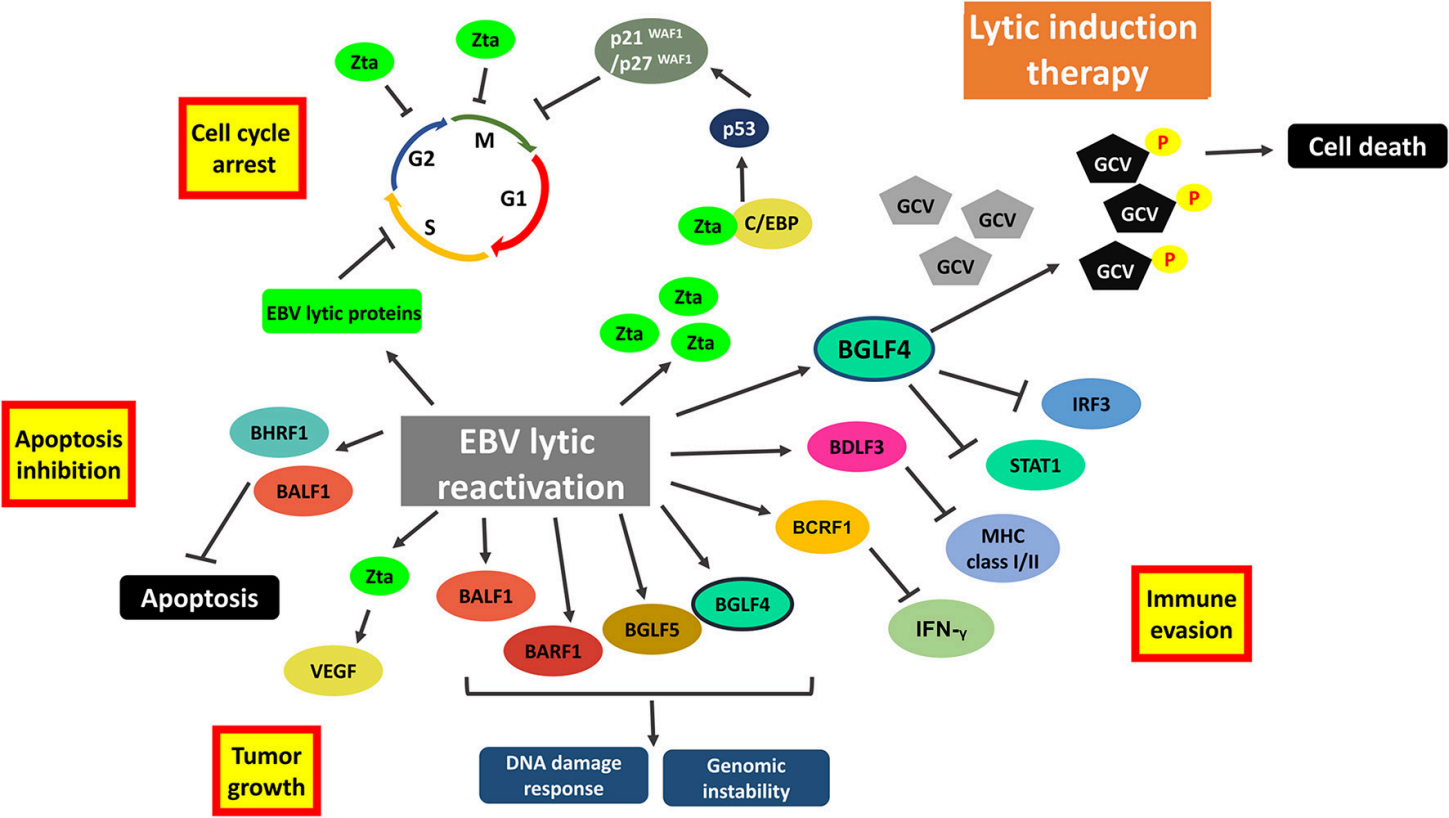
Cellular events associated with EBV lytic reactivation and the rationale of lytic induction therapy. A diverse array of EBV lytic proteins is being expressed during lytic cycle reactivation. Subsequent occurrence of various cellular events include cell cycle arrest, inhibition of apoptosis, tumorigenesis and immune evasion. Expression of viral protein kinase BGLF4 converts antiviral drug e.g., ganciclovir (GCV) from a prodrug to its cytotoxic form, shaping the basis of lytic induction therapy.

Oncolytic therapy which intentionally reactivates lytic cycle of EBV to confer susceptibility of EBV-positive cells to the treatment with antiviral drugs could be a potential therapeutic strategy against EBV-LPD and other EBV-associated diseases. Ganciclovir (GCV), which is a nucleoside-type antiviral drug, is shown to mediate enhanced killing of EBV-positive cancer cells when co-administrated with lytic inducers. This combinatorial strategy relies on the expression of BGLF4, a viral protein kinase expressed during EBV lytic reactivation, to convert GCV into its cytotoxic form (89). The cytotoxic GCV is then incorporated into both viral and cellular DNA of the induced cells and neighboring cells, causing a bystander killing of various EBV-associated malignancies through the induction of premature DNA strand termination and apoptosis in the host cells (83, 90, 91). Alternatively, Fu et al. have demonstrated the possibility of directing [^125^I]2′-fluoro-2′-deoxy-beta-D-5-iodouracilarabinofuranoside ([^125^I]FIAU) to lytically-induced EBV-positive BL cells (92). The lytic induction therapy which employs valporic acid and gemcitabine as lytic inducers has been recently shown to achieve clinical responses in some NPC patients (93, 94). However, some cell populations were refractory to lytic cycle reactivation upon treatment with any of the available lytic inducers (45). Investigating on the lytic reactivation mechanisms by these lytic inducers is essential for developing an effective oncolytic therapy (Figure 7).

### Targeting Survival Pathways in EBV Latency

**Figure 8:**
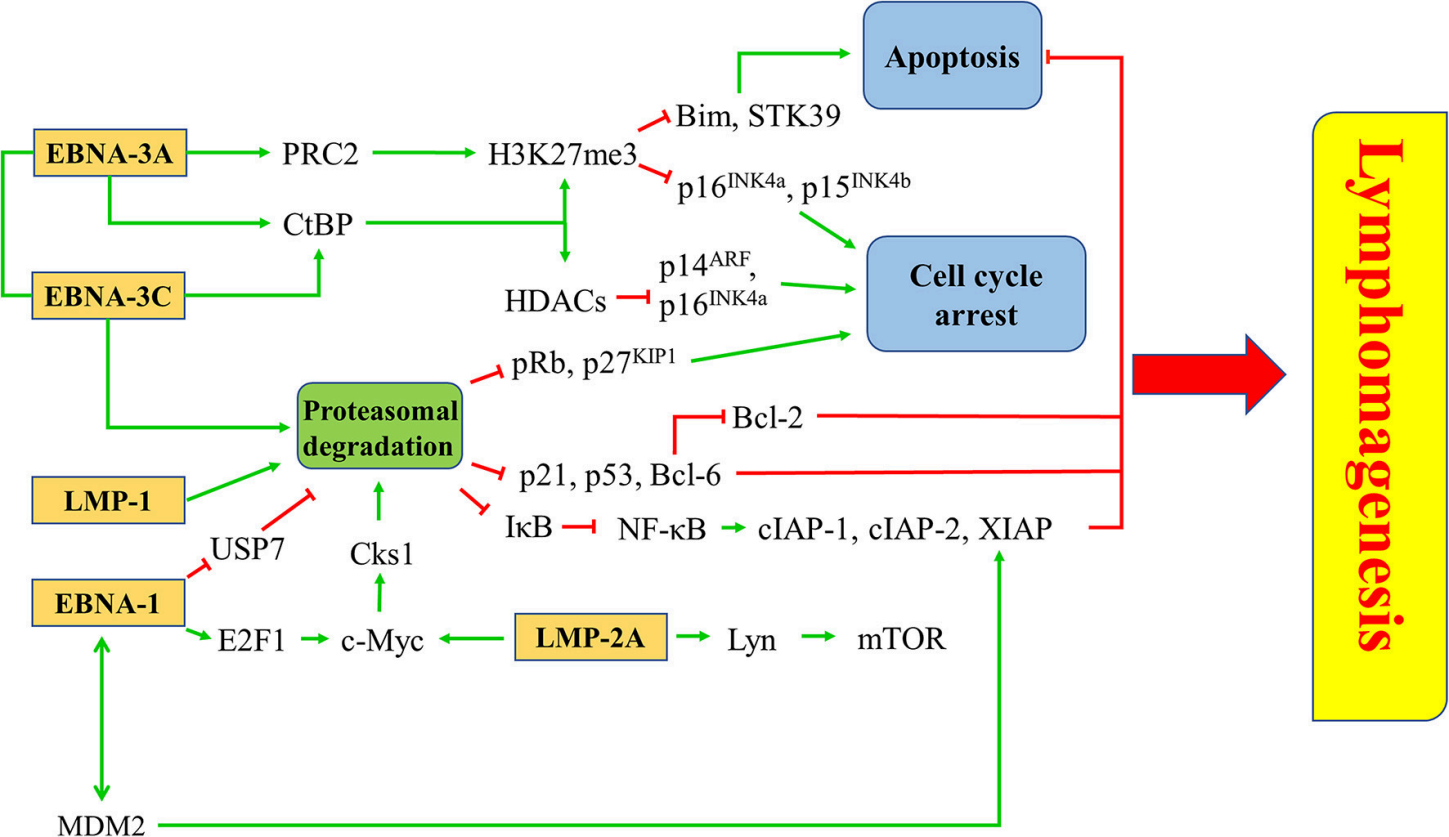
Targeted survival pathways in EBV latency. Several EBV protein-induced survival pathways, such as inhibition of apoptosis and cell cycle arrest through epigenetic repression and/or proteasomal degradation of tumor suppressors for lymphomagenesis can be targeted by novel drugs or drug combinations.

EBV latent proteins, particularly EBNA-1, -3A, -3C, LMP-1, and -2A are shown to be important for the pathogenesis of lymphomas. EBNA-1, which is expressed in all types of EBV-positive cancer cells, represents a specific target for the treatment of EBV-LPDs. A number of inhibitors and vaccines have been developed to target EBNA-1 directly in EBV-positive cancer cells (95–97). MDM2 inhibitors e.g., Nutlin-3a, SAR405838, and JNJ-26854165, or c-Abl kinase inhibitor e.g., Nilotinib, can suppress the growth of lymphomas in EμEBNA-1 transgenic mice via the EBNA-1/MDM2/E2F1 pathway (98). LMP-1 is a CD40 homolog that can activate the NF-κB signaling pathway and exert strong oncogenicity in EBV-LPDs and other EBV-associated malignancies (99, 100). It has been reported that bortezomib, which inhibits the NK-κB signaling, can induce apoptosis in EBV-LPDs and EBV-associated epithelial cancer cells (101, 102). LMP-2A is an EBV-encoded membrane protein which acts as a constitutively active B cell receptor through interacting with Lyn kinase to facilitate B cell transformation and proliferation. Dasatinib is found to inhibit splenomegaly and lymphomagenesis in LMP-2A/MYC double transgenic mice via theinhibition of Lyn (103). Moreover, rapamycin significantly reduces tumor growth, splenomegaly and metastasis of B cell lymphoma through theinhibition of the Lyn-activated mTOR pathway (104). LMP-2A also drives lymphomagenesis through enhancing c-Myc expression which subsequently increases the expression of CDK regulatory subunit 1 (Cks1), a cofactor of the SCF^Skp2^ ubiquitin-ligase complex, leading to the ubiquitination and proteasomal degradation of p27^KIP1^. Proteasome inhibitors, such as MG-132 can reduce lymphomagenesis by increasing the level of p27^KIP1^ (105). EBNA-3 proteins, particularly EBNA-3A and -3C, provide important survival advantages to the EBV-infected cells. For instance, BL cells with type III latency are more resistant to the killing by cytotoxic agents, such as taxol and nocodazole when compare to BL cells with type I latency (106). BL cells with Wp-restricted latency which also express EBNA-3 proteins are more resistant to the killing by anti-IgM or ionomycin when compared to BL cells with type I latency (107). Interestingly, we found that HDAC inhibitors and proteasome inhibitors can act synergistically to induce the up-regulation of p21^WAF1^ and mediate enhanced killing to the BL cells with Wp-restricted or type III latency but not to those with type I latency, suggesting the involvement of EBNA-3 proteins in the cell death mechanism (101). We further tested the mechanism of killing of BL cell lines infected with EBNA3A, -3B, or -3C knockout EBV or with their revertant EBV and found that EBNA3C-expressing cells can bypass the G2/M checkpoint arrest induced by the combination of HDAC and proteasome inhibitors and subsequently become more susceptible to the induction of apoptosis (108). Such enhanced killing is due to the up-regulation of p21^WAF1^ and down-regulation of p-cdc25c in EBNA3C-expressing cells (108) (Figure 8).

## Novel Drugs or Drug Combinations Targeting EBV Latent and Lytic Cycles

**Figure 9:**
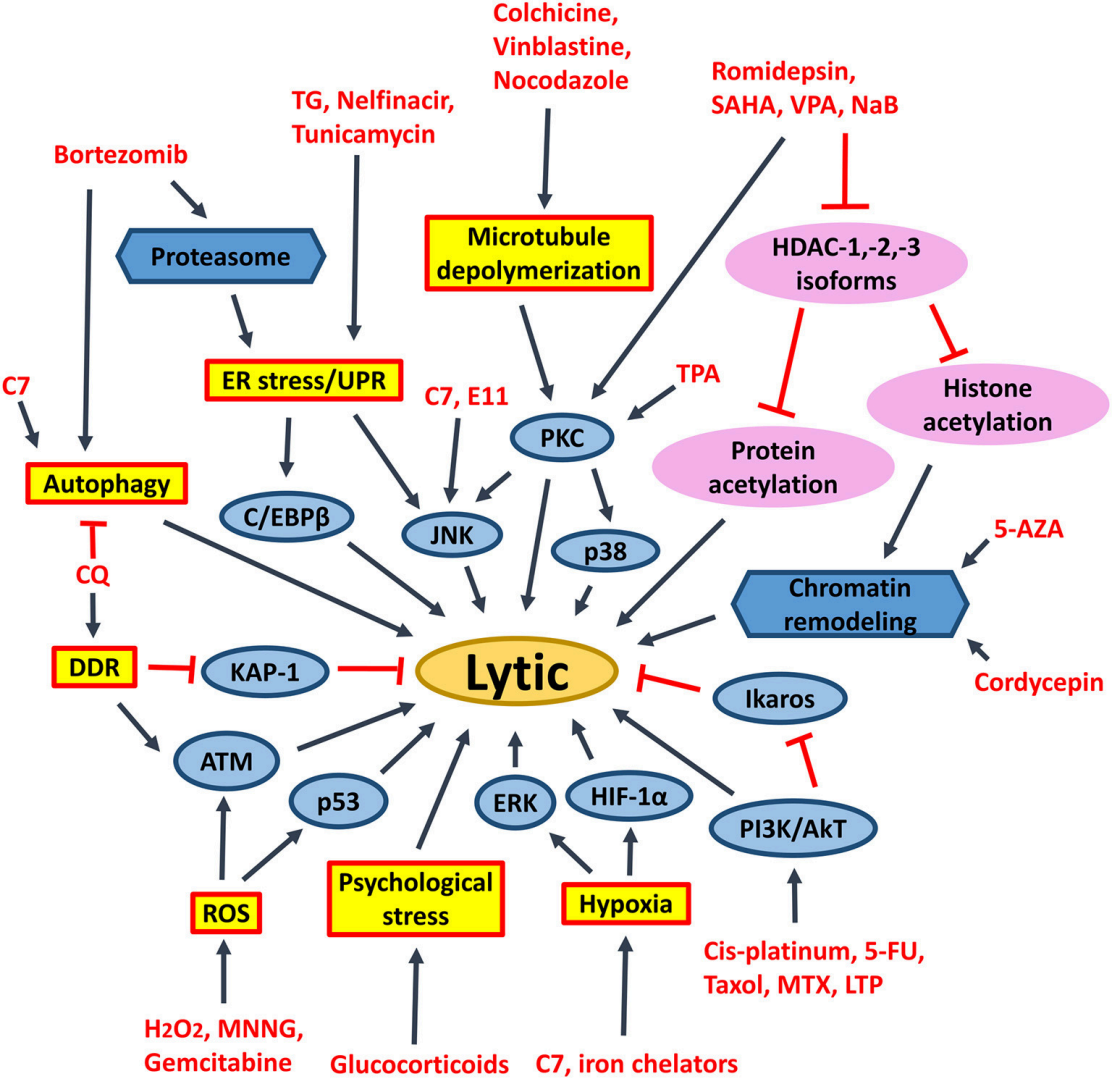
Signaling pathways activated by different chemical lytic inducers for EBV lytic reactivation. EBV lytic reactivation can be achieved through the activation of different cellular signaling pathways e.g., PKC, p38/MAPK, ERK1/2, JNK, PI3K/AKT, DDR, ROS, hypoxia, ATM signaling pathways as well as inhibition of Ikaros and chromatin remodeling. 5-FU, fluorouracil; MTX, methotrexate; 5-AZA, 5-azacytidine; SAHA, suberoylanilide hydroxamic acid; TPA, 12-O-tetradecanoylphorbol-13-acetate; CQ, chloroquine; PKC, protein kinase C; p38/MAPK, P38 mitogen-activated protein kinases; ERK1/2, extracellular signal-regulated protein kinases 1 and 2; JNK, c-Jun N-terminal kinase; C/EBP, CCAAT/enhancer binding proteins; PI3K/AKT, phosphatidylinositol 3-kinase/AKT; TG, thapsigargin; MNNG, methylnitronitrosoguanidine; DDR, DNA damage response; ROS, reactive oxygen species; ER stress, endoplasmic reticulum stress; UPR, unfolded protein response.

**Figure 10:**
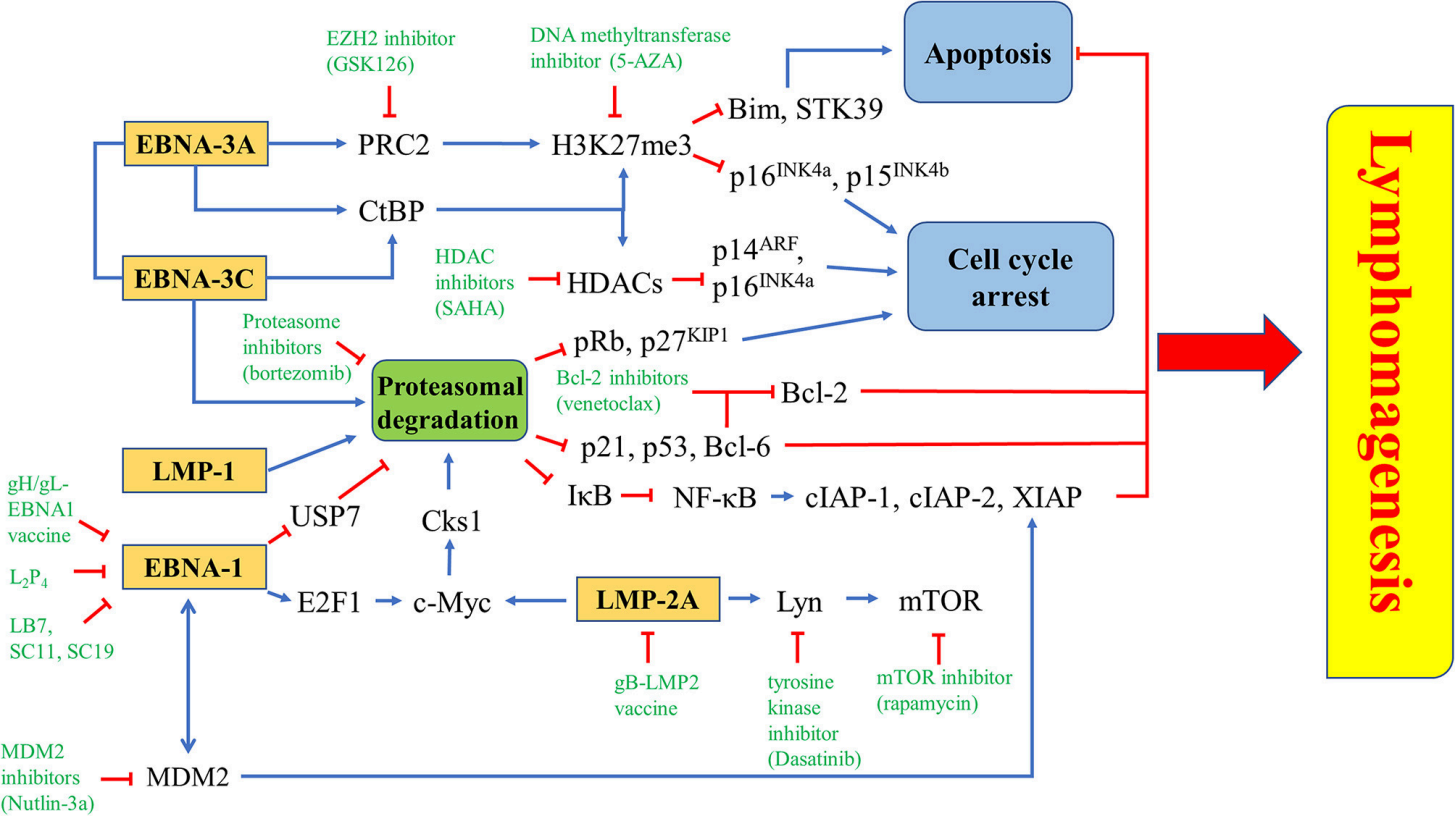
Novel drugs or drug combination targeting EBV latency. EZH2 inhibitior (GSK126), DNA methyltransferase inhibitor (5-AZA) or HDAC inhibitor (SAHA) is used to inhibit the epigenetic repression of Bim, STK39, p14^ARF^, p15^INK4b^, and p16^INK4a^ triggered by EBNA-3A and/or EBNA-3C. Proteasome inhibitor (bortezomib) can inhibit proteasomal degradation of tumor suppressors induced by EBNA-1, EBNA-3A, EBNA-3C, LMP-1, or LMP-2A. There are some EBNA-1 inhibitors, including gH/gL-EBNA1 vaccine, L_2_P_4_, LB7, SC11 and SC19, while gB-LMP2 vaccine is found to inhibit LMP-2A. Several downstream molecules, such as Lyn, mTOR, and MDM2 can be targeted by tyrosine kinase inhibitor (Dasatinib), mTOR inhibitor (rapamycin), and MDM2 inhibitor (Nutlin-3a), respectively.

Several FDA-approved drugs and novel compounds are shown to have effects on either inducing EBV lytic cycle or targeting the survival mechanisms delivered by the EBV latent proteins in EBV-positive cancer cells. These compounds work in diverse mechanisms including inhibition of HDAC and proteasome, activation of MAPK pathways, induction of various cellular stress responses (e.g., ER stress, DNA damage response, hypoxia and oxidative stress), autophagy, cell cycle arrest and apoptosis (Table 1, Figures 9, 10).

**Table 1:** Summary on the therapeutic strategies and their corresponding molecular mechanisms against EBV-associated LPDs.

**Therapeutic strategy**	**Method**	**Molecular mechanism**
Gene Therapy	Thymidine kinase (70) p53 delivery (71)	To deliver cytotoxic proteins or chemosensitizers to EBV-infected malignancies that induce apoptosis or enhance sensitivity to GCV
Immunotherapy	Virus-specific cytotoxic T lymphocytes (CTLs)	CTLs that target EBV latent proteins are isolated from patients are infused back to patients after activation and expansion of T lymphocytes *in vitro* (73, 74)
	Virus-like particles (VLPs) vaccines	Induction of neutralizing antibody titres against EBV via immunization with VLP vaccines e.g., gH/gL-EBNA-1 and bG-LMP2 without adjuvant (79)
Lytic induction therapy	Histone deacetylase (HDAC) inhibitors (e.g., VPA, SAHA, romidepsin)	Reactivation of EBV lytic cycle through the activation of PKC-δ and ATM signaling pathway (8, 43, 89, 109)
	Proteasome inhibitors (e.g., bortezomib)	EBV lytic reactivation via the activation of ER stress, CCAAT/enhancer-binding protein β (C/EBPβ), JNK and autophagy (110–112)
	ER stress inducers (e.g., thapsigargin (TG), tunicamycin, Bortezomib, nelfinavir)	EBV lytic reactivation via the induction of ER stress and UPR (111, 113)
	Psychological stress inducers (e.g., hydrocortisone, dexamethasone)	EBV lytic reactivation via the activation of Z promoter specifically (114)
	DNA damage inducers (e.g., chloroquine)	EBV lytic reactivation via the activation of ATM and phosphorylation of KAP1/TRIM28 (115)
	Microtubule depolymerisation (e.g., colchicine, vinblastine, nocodazole)	EBV lytic reactivation via the activation of PKC and the downstream p38 MAPK and JNK signaling pathways (116)
	Hypoxia induction (e.g., iron chelators, C7)	EBV lytic reactivation via the stabilization of HIF-1α which directly binds to the Z promoter; and the induction of ERK-autophagy axis (117)
	ROS activation (e.g., MNNG)	EBV lytic reactivation via the activation of ATM, p38 MAPK and JNK signaling pathways (118)
	Genotoxic stress (e.g., gemcitabine)	EBV lytic reactivation via the activation of ATM and p53 signaling pathways (90, 93, 119)
	Chemotherapeutic agents (e.g., gemcitabine, doxorubicin)	EBV lytic reactivation via the activation of PI3K, p38 MAPK and MEK signaling pathways (93)
	Immunosuppressive drugs (e.g., methotrexate)	EBV lytic reactivation via the activation of p38 MAPK, PI3K and ERK signaling pathways (120)
	Immunomodulatory agents (e.g., lenalidomide, thalidomide, pomalidomide)	EBV lytic reactivation via the activation of PI3K and suppression of Ikaros (121)
Targeting survival pathways in EBV latency	MDM2 inhibitors (e.g., nutlin-3a, SAR405838, JNJ-26854165)	Suppress the growth of lymphoma via the EBNA-1-MDM2-E2F1 pathway (99)
	c-Abl kinase inhibitors (e.g., nilotimib)	Suppress the growth of lymphoma via the EBNA-1-MDM2-E2F1 pathway (99)
	Lyn inhibitors (e.g., dasatinib)	Inhibit splenomegaly and lymphomagenesis via Lyn inhibition (104)
	mTOR inhibitors (e.g., rapamycin)	Reduce tumor growth, splenomegaly and metastasis via mTOR inhibition (105)
	EZH2 inhibitors, DNA methyltransferase inhibitors	Induction of cell cycle arrest via the inhibition of the catalytic subunit of PRC2 as well as histone methylation (16, 30, 36)
	Proteasome inhibitors (e.g., bortezomib, MG-132)	Induction of cell cycle arrest via upregulation of p21^WAF1^ and p27^KIP1^ (122–124)
	Combination of proteasome inhibitors and HDAC inhibitors (e.g., bortezomib and SAHA)	Induction of G2/M arrest and apoptosis via the generation of ROS, upregulation of p21^WAF1^ and p27^KIP1^ (102)

### Histone Deacetylase (HDAC) Inhibitors

HDAC inhibitors of different selectivity are developed to inhibit the actions of various HDAC isoforms in the human cells. Pan-HDAC inhibitors inhibit class I (i.e., HDAC-1/-2/-3/-8), class II (i.e., HDAC-4/-5/-6/-7/-9/-10), and class IV (i.e., HDAC-11) but not class III HDAC isoforms (125). We have shown that several pan-HDAC inhibitors, such as sodium butyrate, valproic acid and SAHA can induce EBV lytic reactivation in EBV-associated epithelial cells (11, 88). We have further demonstrated that selective inhibition of HDAC-1, -2, and -3 by siRNA or specific HDAC inhibitors (e.g., romidepsin, MS-275 and apicidin) is sufficient to reactivate EBV lytic cycle and mediate enhanced killing with ganciclovir *in vitro* and *in vivo* (45) (Figure 9). Inhibition of HDAC-2 and -3 can also reactivate the lytic cycle of human immunodeficiency virus (HIV) and Kaposi's sarcoma-associated herpesvirus (KSHV) (126–128). SAHA and romidepsin are FDA-approved drugs for treating several types of malignancies, such as peripheral T-cell lymphoma and cutaneous T-cell lymphoma (129). Both drugs are able to induce EBV lytic cycle in concentrations that are acceptable in the plasma of patients (45, 88). As mentioned in the previous section, the lytic induction therapy that employs valproic acid and gemcitabine as lytic inducers is found to be safe and feasible for the treatment of NPC patients in clinical trials (93, 94). We postulate that substitution of valproic acid with either romidepsin or SAHA would probably lead to a better treatment effect because SAHA or romidepsin has a higher potency in reactivating the EBV lytic cycle *in vitro* (11, 45).

It was believed that acetylation of histones on Z and R promoters is responsible for the effect of HDAC inhibitors on the reactivation of EBV lytic cycle (130). However, several reports have shown that acetylation of histones alone is not sufficient for EBV lytic cycle reactivation (131–134). Our laboratory has also shown that proteasome inhibitors work synergistically with HDAC inhibitors to induce histone acetylation, but simultaneously suppress the reactivation of EBV lytic cycle in epithelial cells mediated by HDAC inhibitors (102, 135). We postulate that acetylation of non-histone proteins, rather than histone proteins, could directly regulate the reactivation of EBV lytic cycle upon treatment with HDAC inhibitors. The transcription factors which bind to Z promoter, including CREB, C/EBP, Sp1, Sp3, MEF2D, YY1, ZEB1/2, can be modified by lysine acetylation (136–142). It is possible that acetylation of these transcription factors would directly affect their activities on the Z promoter (Figure 9).

### Proteasome Inhibitors

Proteasome inhibitors can either covalently or non-covalently bind to 20S proteasome which catalyzes the degradation of ubiquitinated proteins (143). They can induce unfolded protein response (UPR), endoplasmic reticulum (ER) stress, reactive oxygen species (ROS) generation, upregulation of p21^WAF1^ and p27^KIP1^, which subsequently lead to cell death in a variety of cancer types (144–146).

Whilst EBNA-1, LMP-2A and LMP-1 inhibit the proteasomal degradation pathway for maintaining viral latency; EBNA-3C utilizes the proteasome system to promote proteasomal degradation of tumor suppressors (e.g., pRb, p21^WAF1^, p27^KIP1^, p53 and Bcl-6) which regulate the cell cycle and apoptosis in the host cells (26, 28–30, 54, 122–124, 143, 147, 148). In our previous study, we have found that bortezomib can induce cell cycle arrest at G2/M phase with a higher percentage of BL cells when compared with LCLs which express a higher level of EBNA-3C protein (101). We have further demonstrated that when EBNA-3C knockout or EBNA-3C revertant BL cells are treated with bortezomib, there is a G2/M arrest in the EBNA-3C knockout cell lines whilst the G2/M arrest is bypassed in the EBNA-3C revertant cell lines (108). In these studies, bortezomib in combination with SAHA can induce a stronger apoptotic effect in the EBNA-3C revertant than EBNA-3C knockout BL cells (101). We postulate that disruption of survival signaling conferred by EBNA-3C, such as the suppression of p53, p21^WAF1^ and Bcl-6 might be responsible for the induction of apoptosis (54–56). In addition, bortezomib can also induce EBV lytic cycle in BL cells (149, 150). The mechanism of lytic induction by bortezomib is mediated via the activation of ER stress, C/EBP-β, JNK and autophagy (150, 151). However, the effect of bortezomib on EBV lytic cycle reactivation is limited to BL cells but not EBV-positive epithelial cancer cells (102, 152) (Figure 10).

### Stress Inducers

Endoplasmic reticulum (ER) stress inducers, including hapsigargin (TG), tunicamycin, bortezomib and nelfinavir, are shown to induce EBV lytic cycle via the induction of ER-stress or UPR (110, 150). Induction of psychological stress by hydrocortisone and dexamethasone can also activate the Z promoter in BL cells (111). Induction of DNA damage response by chloroquine can reactivate EBV lytic cycle via the activation of ATM and phosphorylation of KAP1/TRIM28 in BL cells (112). Activation of ATM is further shown to be essential for the lytic cycle reactivation by conventional lytic inducers, such as HDAC inhibitors, transforming growth factor β (TGF-β) and anti-IgG in several EBV-positive cell lines (153). Induction of microtubule depolymerization by colchicine, vinblastine and nocodazole can reactivate EBV lytic cycle through the activation of PKC and the downstream p38 MAPK and JNK signaling pathways in NPC cells (113). Induction of hypoxia by iron chelators is reported to reactivate EBV lytic cycle through the direct binding of HIF-1α to the HRE elements within Z promoter (114). Generation of ROS upon treatment with methylnitronitrosoguanidine (MNNG) can induce EBV lytic cycle through the activation of ATM, p38 MAPK and JNK signaling pathways (115). Activation of the ATM/p53 genotoxic stress pathway by gemcitabine is found to induce the lytic cycle of EBV in BL, gastric carcinoma and NPC cells (90, 93, 109). TPA in combination with sodium butyrate activates PCK-θ and the subsequent p38 MAPK pathway to reactivate the EBV lytic cycle (116). Chemotherapeutic agents including gemcitabine and doxorubicin can reactivate the lytic cycle of EBV in BL cells and LCLs via the activation of PI3K, p38 MAPK, and MEK (90). An immunosuppressive drug, methotrexate (MTZ), is also shown to induce EBV lytic cycle via the activation of PI3K, p38 MAPK, and ERK signaling pathways in EBV-positive lymphoma cells (117). Immunomodulatory agents, including lenalidomide, thalidomide and pomalidomide reactivate the lytic cycle of EBV in EBV-positive BL cells via PI3K stimulation and ikaros suppression (118) (Figure 9). Upon induction of these stress signaling pathways and the subsequent reactivation of EBV lytic cycle, increased cell death is concomitantly observed (11, 90, 93, 109, 117, 118).

### Induction of Autophagy

Activation of ERK and autophagy by Rta is shown to be essential for the EBV lytic progression in BL cells (119). Induction of autophagy via the activation of PKC-θ and p38 MAPK is also demonstrated to be essential for the reactivation of EBV lytic cycle in B cells upon treatment with the combination of TPA and sodium butyrate (116). Granato et al. have shown that reactivation of EBV lytic cycle by bortezomib also requires autophagy initiation (151). Recently, chloroquine is shown to activate ATM to phosphorylate KAP1/TRIM28, which is normally involved in repairing double-strand breaks in heterochromatin, to reactivate EBV lytic cycle in BL cells (112). However, the lytic proteins expressed in early phase of EBV lytic cycle will block autolysosome formation to prevent viral degradation and enhance the viral replication in B cells (120, 121). We have recently demonstrated that a novel compound C7 can reactivate EBV lytic cycle via an autophagy-dependent mechanism in EBV-associated epithelial cells (154) (Figure 9). On the other hand, constitutive activation of autophagy is found to confer resistance to nutlin-3 induced apoptosis in the BL cells with type III latency but not in the BL cells with type I latency, suggesting a possible role of EBV latent proteins in the regulation of autophagy (155).

### Induction of Cell Cycle Arrest

EBNA-3A, together with EBNA-3C, can recruit polycomb repressor complex 2 (PRC2) or interact with C-terminal Binding Protein (CtBP) to epigenetically down-regulate tumor suppressor genes. Transcription of cell cycle regulatory factors, such as p14^ARF^, p16^INK4a^, and p15 ^INK4b^, are repressed by methylation and deacetylation of histones mediated by EBNA-3A and EBNA-3C (32, 156). Treatment with enhancer of zeste homolog 2 (EZH2) inhibitors and DNA methyltransferase inhibitors, which inhibit the catalytic subunit of PRC2 and histone methylation, might probably induce cell cycle arrest in EBV-LPDs. EBNA-3A and -3C are known to directly interact with HDAC-1 and -2 to repress the expression of p14^ARF^ and p16^INK4a^ (32, 38, 157). EBNA-3C is also shown to recruit Pim-1 to phosphorylate and suppress pRb, p21^WAF1^ and p27^KIP1^ via the proteasomal degradation system in B cells (26, 28, 29). Interestingly, we have shown that combination of proteasome and HDAC inhibitors can upregulate p16^INK4a^ and p21^WAF1^, and mediate G2/M arrest in EBV-LPDs (101). The G2/M arrest is further demonstrated to be related to the downregulation of p-cdc25c (108) (Figure 10).

### Induction of Apoptosis

LMP-1 can activate both canonical (acts through the p50/RelA dimer) and non-canonical (acts through the p52/RelB dimer) NF-κB pathways to promote the pathogenesis of EBV-positive cancer cells (158, 159). Bortezomib can inhibit the proteasomal degradation of IκBα, thus, inhibit the activation of NF-κB pathways. Consequently, the expression of anti-apoptotic proteins including X-chromosome-linked inhibitor-of-apoptosis protein (XIAP), cellular inhibitor-of-apoptosis protein 1 (cIAP-1) and c-IAP-2, are suppressed (149). EBNA-3A and -3C can work together to epigenetically down-regulate tumor suppressor genes including STK39 and Bim to inhibit apoptosis (58, 160–162). It has been demonstrated that the EZH2 inhibitor GSK126 can inhibit the PRC2 complex and increase the expression of STK39 whereas the DNA methyltransferase inhibitor 5-AZA can significantly increase the mRNA level of STK39 in LCLs (160). The EZH2 inhibitor can also significantly induce the expression of Bim and apoptosis in BL cells (162). EBNA-3C promotes proteasomal degradation of certain tumor suppressors, such as p21^WAF1^, p53, and Bcl-6 (28, 30, 54). We have reported that combining HDAC and proteasome inhibitors can upregulate p21^WAF1^ and mediate synergistic killing of BL cells and LCLs via an EBNA-3C-dependent mechanism (101, 108). As inhibition of Bcl-6 by EBNA-3C releases Bcl-2 to suppress apoptosis, it is possible that combination of Bcl-2 inhibitors, such as venetoclax and proteasome inhibitors, such as bortezomib could synergistically induce apoptosis in EBV-LPDs (Figure 10).

### Novel Compounds Specifically Target EBV

The abovementioned treatment strategies, such as HDAC inhibitors, proteasome inhibitors and other stress inducers could all affect multiple signaling pathways and thus, resulting in non-specific effects to the host cells. Identification of novel compounds which can specifically target EBV latent and lytic cycles is critical for further development of viral-targeted therapy against EBV-LPDs. Using computational docking programs, Li et al. have identified 4 structurally related compounds (coded SC7, SC11, SC19 and SC27) which can inhibit the DNA binding of EBNA-1 and reduce the viral genome copy in BL cells (96). In a separate study, the same research team has identified 4 additional EBNA-1 specific inhibitors (coded LB2, LB3, LB7 and LC7) via a cell-based screening of 14,000 small molecule compounds (97). Recently, a peptide-based inhibitor, L_2_P_4_, which can bind to EBNA-1 and inhibit EBNA-1 homodimer formation is found to selectively inhibit the proliferation of EBV-positive BL and NPC cells *in vitro* and *in vivo* (95) (Figure 10). Tikhmyanova et al. have identified 5 structurally related tetrahydrocarboline derivatives coded C09, C50, C53, C60, and C67, which can significantly reactivate the lytic cycle of EBV and mediate enhanced killing with GCV in both lymphoma and epithelial cancer cells from a high-throughput screening of 66,840 small molecule compounds (163). These compounds do not induce acetylation of histone and phosphorylation of p38 MAPK, S6, p53, and p90RSK, suggesting a distinct lytic reactivation mechanism from those induced by HDAC inhibitors or TPA (163). Our laboratory has also identified 5 hit compounds (coded C7, E11, E7, C8, and A10) through a high-throughput screening of 50,240 small organic compounds (164). These compounds also do not phosphorylate the PKCδ which is utilized by many conventional lytic inducers and do not cause acetylation of histone, suggesting a mechanism of action distinct from HDAC inhibitors or TPA (164). E11 and C7 are further investigated for their mechanisms of EBV lytic cycle reactivation. We have found that the lytic cycle reactivation by E11 requires the JNK signaling pathway whilst the lytic cycle reactivation by C7 requires the activation of both ERK and JNK pathways (164). We have further demonstrated that C7 can reactivate EBV lytic cycle in epithelial cells via chelation of iron and induction of autophagy (154). Recently, another new class of lytic inducer, curcuminoids, which might reactivate EBV lytic cycle through modulation of NK-κB signaling, is identified (165). Since most of these novel compounds can reactivate EBV lytic cycle via mechanisms distinct from conventional inducers, such as HDAC inhibitors and TPA, it would be interesting to test whether combination of conventional inducers with novel inducers can mediate synergistic reactivation of EBV lytic cycle in EBV-LPDs (Figure 9).

## Conclusions

In this review, we have summarized the mechanisms by which EBV-LPDs employ to drive the pathogenesis and maintain the survival of the tumor cells. We have shown that the viral latent and lytic proteins can maintain the proliferation and survival of EBV-positive tumor cells via deregulating the mechanisms which control the cell cycle, apoptosis and immune recognition in the host cells. Potential viral-targeted strategies based on the understanding of the patho-mechanisms of EBV-LPDs are also discussed. A number of clinical relevant drugs and novel compounds which can either target the EBV latent proteins or reactivate EBV lytic cycle are reviewed. These compounds work through diverse mechanisms including inhibition of HDAC and proteasome, activation of MAPK pathways, induction of various cellular stress responses (e.g., ER stress, DNA damage response, hypoxia and oxidative stress), autophagy, cell cycle arrest and apoptosis. The effect of combining pharmaceutic compounds which act on multiple signaling pathways in EBV-LPDs should be explored.

## Author Contributions

AC conceived the project. KH and AC wrote the manuscript with the help of SY and KT.

### Conflict of Interest Statement

The authors declare that the research was conducted in the absence of any commercial or financial relationships that could be construed as a potential conflict of interest.
